# Learning a Foreign Language in Older Adults Shapes the Functional Connectivity of Distinct Cerebellar Sub‐Regions With Cortical Areas Rich in CB_1_ Receptor Expression

**DOI:** 10.1002/brb3.70565

**Published:** 2025-05-26

**Authors:** Giovanna Bubbico, Federica Tomaiuolo, Carlo Sestieri, Golnoush Akhlaghipour, Alberto Granzotto, Antonio Ferretti, Mauro Gianni Perrucci, Stefano L. Sensi, Stefano Delli Pizzi

**Affiliations:** ^1^ Department of Neuroscience, Imaging and Clinical Sciences “G. d'Annunzio” University of Chieti‐Pescara Chieti Italy; ^2^ Institute for Advanced Biomedical Technologies (ITAB) “G. d'Annunzio” University of Chieti‐Pescara Chieti Italy; ^3^ Department of Engineering and Geology “G. d'Annunzio” University of Chieti‐Pescara Chieti Italy; ^4^ Department of Neurology Montefiore Einstein Saul R. Korey Bronx New York USA; ^5^ Centre for Advanced Studies and Technology (CAST) “G. d'Annunzio” University of Chieti‐Pescara Chieti Italy; ^6^ UdA‐TechLab, Research Center “G. d'Annunzio” University of Chieti‐Pescara Chieti Italy; ^7^ Neurology Institute SS Annunziata University Hospital, “G. d'Annunzio” University of Chieti‐Pescara Chieti Italy

**Keywords:** aging, cannabinoid, cerebellum, fMRI, foreign language learning, GABAa

## Abstract

**Background:**

Foreign language learning (FLL) in older adults is a comprehensive cognitive enhancement tool that integrates linguistic, cognitive, and social components to stimulate neuroplasticity and promote brain reorganization to counteract age‐related decline. While previous studies have investigated the impact of FLL on the cortical connectome, its effects on subcortical‐cortical resting‐state functional connectivity (rs‐FC) remain unexplored. The present study focuses on the connectivity of the cerebellum, based on its involvement in learning and aging. We hypothesize that FLL primarily modulates the rs‐FC of the most “cognitive” cerebellar sub‐regions, such as the crus and the posterior lobules.

**Methods:**

The rs‐FC pattern was analyzed in 27 participants who underwent a 4‐week FLL (*n* = 14) or a control (*n* = 13) protocol. Using distinct cerebellar regions as seeds in voxel‐wise analyses, we evaluated FLL‐induced changes in cerebellar–neocortical connectivity. Furthermore, we quantitatively assessed the spatial overlap between the connectivity modulations and the expression of neurotransmitter receptors associated with neuroplasticity, using data from publicly available repositories.

**Results:**

The FLL group showed distinct changes in cerebellar‐neocortical rs‐FC, including reduced connectivity between Crus I/Vermis IV‐V and the visual cortex and increased connectivity between Lobule VI/VIIb and frontal regions. The connectivity changes involving Crus I and Lobule VI spatially overlapped with the distribution of CB1 receptors and, to a lesser extent, between the connectivity changes of Crus I/Lobule VI and Vermis IV‐V and mGluR5/GABAa receptors.

**Conclusions:**

We provide new insights into the involvement of the cerebellum in the beneficial effects of FLL in aging, further highlighting the role of CB1 receptors and, secondarily, mGluR5/GABAa receptors.

## Graphical abstract

Learning a foreign language in older adults altered the connectivity of Crus I and Lobule VI with associative and sensory areas, overlapping the distribution of CB1 receptors.

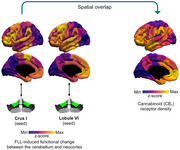

## 1 Introduction

Foreign language learning (FLL) in older age is considered a promising cognitive enhancement strategy for maintaining cognitive reserve and promoting brain plasticity to compensate for cognitive decline during aging (Venugopal et al. 2024). Unlike other forms of cognitive training, FLL combines linguistic, cognitive, and social aspects, offering a unique multifactorial approach (Chai et al. 2016; Jafari et al. 2021; Stein et al. 2012; Voits et al. 2024). Managing the cognitive demands of handling multiple languages—such as switching between language systems, inhibiting interference, and monitoring speech—requires a continuous combination of mechanisms underlying executive control, attention, and saliency detection (Bialystok et al. 2004; Emmorey et al. 2008; Prior and Gollan 2011). Neuroimaging studies using functional fMRI during tasks and rest states, including our previous work (Bubbico et al. 2019), have shown that, in older adults, FLL modulates global and localized network architecture (Kliesch et al. 2022) as well as brain activity and functional connectivity in frontoparietal areas (Schultz et al. 2024; Teubner‐Rhodes et al. 2017; Wingfield and Grossman 2006). These cortical areas are part of the dorsal attention (e.g., superior parietal cortex), the default mode (DMN) (e.g., superior frontal cortex), the frontoparietal control (e.g., inferior frontal cortex) (Bubbico et al. 2019), and the salience network (e.g., cingulo‐opercular cortex) (Teubner‐Rhodes et al. 2017).

In contrast, the effect of FLL on the connectivity of subcortical structures, especially those that have been previously associated with both learning and aging, remains unexplored. For example, the cerebellum regulates cognitive functions through its interactions with several cortical associative areas (Habas et al. 2009), contributing to shaping intrinsic connectivity networks such as the DMN, the frontoparietal control network (FPCN), and the salience and attentional networks. Neuroimaging and clinical studies highlight the cerebellum's role in various language tasks and linguistic deficits following cerebellar disease (Marien and Borgatti 2018). Functional imaging reveals that cerebellar lesions can impact distant cortical areas through crossed cerebellar–cerebral diaschisis (Marien and Borgatti 2018). Evidence indicates that the cerebellum, via its lateralized and reciprocal connections with the cortex, contributes to language processing by coordinating cortical functions through timing and sequencing mechanisms, akin to its role in motor control (Marien and Borgatti 2018). Disruptions in specific cerebellar–neocortical networks have been associated with aging (Bernard et al. 2013) and neurodegenerative diseases, with alterations in cerebellar circuits observed, for example, in the DMN in Alzheimer's disease and the salience network in frontotemporal dementia (Guo et al. 2016).

From an anatomo‐functional perspective, cerebellar regions are gradually organized, progressing from primary to heteromodal areas. While its anterior portions are primarily involved in motor coordination, more posterior regions—such as the crus I‐II and lobules VI‐X—are associated with learning, memory, attentional processing, and executive functions (Xue et al. 2021). Therefore, posterior cerebellar regions may play a pivotal role in the reorganization of brain networks observed during FLL in aging. Studies have shown that inputs from the cerebellum to the dorsal attention network (DAN) activate the attentional system (Brissenden et al. 2016). This cerebellar influence increases with age and is inversely correlated with processing speed (Arleo et al. 2024). The neural substrates underlying these processes are located in lobule VI of both hemispheres and the cerebellar vermis—a region known for its cognitive role in supporting timing, perception, and task automatization (Begue et al. 2024).

Based on these findings, our working hypothesis is that FLL should modulate the connectivity of the more “cognitive” cerebellar regions, such as the crus and posterior lobules, which are the cerebellar counterparts of cortical networks previously associated with the effect of FLL. To test this hypothesis, we analyzed blood‐oxygenation‐level‐dependent (BOLD) fMRI data in a sham‐controlled study, which included a cohort of 27 healthy older adults who underwent a 4‐week FLL (*n* = 14) or a sham protocol (*n* = 13). Using seed‐based analyses, one for each of the 25 cerebellar subregions defined by the anatomical labeling atlas 3 (AAL‐3; Rolls et al. 2020), we examined the resting‐state functional connectivity (rs‐FC) changes induced by the FLL protocol on the cerebellar–neocortical connectivity. The strength of rs‐FC changes was correlated with neuropsychological test scores to determine whether the identified modulations of rs‐FC were associated with the benefit of FLL in specific cognitive domains. Finally, a network‐level analysis investigated the spatial overlap between the effects of FLL on brain connectivity and the expression of receptors that have been closely associated with neuroplasticity in aging, such as the CB_1_ (Bilkei‐Gorzo 2012; Bilkei‐Gorzo et al. 2017), GABA_a_ (Rissman et al. 2007; Rissman and Mobley 2011), NMDA (Hunt and Castillo 2012), and mGluR5 (Carvalho et al. 2019).

## 2 Experimental Procedures

This study re‐analyzes data from Bubbico et al. (2019) with new methodological advancements and additional participants in the sham groups. It explores the impact of FLL on connectivity between cerebellar regions and the neocortex and represents the first investigation of the spatial correlation between these effects and the expression of cortical receptors involved in neuroplasticity. The study was approved by the Research and Ethics Committee (ethical approval number: Prot. Nr. 835, 24‐04‐2015), with all participants providing written informed consent. All procedures followed the ethical guidelines of the Declaration of Helsinki.

### 2.1 Experimental Design

A total of 27 older adults, randomly assigned to the two groups using a 1:1 ratio, completed the study. A number of 13 participants were included in the Sham group and 14 in the FLL group.

The FLL group participated in a structured 16‐week beginner‐level English language course, consisting of a weekly 2‐h session (32 total hours), delivered in person in small groups of four to six participants to encourage individual engagement and interaction. Each session comprised two consecutive 45‐min classes taught by a certified native English‐speaking instructor, separated by a 15‐min break, and was complemented by approximately 30 min of weekly homework. The course followed a progressive curriculum designed for older adult learners and aimed to build foundational English language skills. Instruction focused on basic vocabulary and grammar, including sentence structure, verb conjugation, prepositions, and commonly used expressions relevant to everyday contexts such as health, transportation, shopping, and food. Listening and speaking skills were developed through dialogues, role‐playing exercises, and group discussions to promote oral comprehension and verbal fluency, while reading and writing were practiced through short texts, sentence‐completion activities, and the production of simple narratives describing personal experiences or daily routines. Cultural aspects of British and American English, such as traditions, holidays, and idiomatic expressions, were introduced to strengthen socio‐pragmatic competence. Weekly team‐based projects, including planning imaginary trips or preparing short group presentations, were used to promote active language use and collaborative learning. Participants were also encouraged to maintain a weekly language diary to reflect on their progress and were given access to optional supplementary materials such as audio recordings and word games. A qualitative assessment conducted at the beginning and end of the course evaluated individual progress in vocabulary acquisition, grammatical accuracy, pronunciation, and communicative confidence.

The control group did not participate in any training but completed the same pre‐ and post‐tests. To ensure consistency, the participants received monthly phone calls to confirm that they maintained their usual lifestyle during the 4‐month study period.

Our prior publications have outlined comprehensive details regarding the study's design, patients' clinical histories, and inclusion/exclusion criteria (Bubbico et al. 2019).

### 2.2 Cognitive Assessment

All participants completed a neuropsychological battery before and after the FLL intervention. This assessment measured various cognitive domains, including executive functions, working memory, short‐term and long‐term verbal memory, and fluid intelligence. Global cognition decline was assessed using the Mini‐Mental State Examination (MMSE). Participants completed the Trail Making Test (TMT) A for sustained spatial attention, TMT B for divided spatial attention, and TMTB‐A for cognitive flexibility. The verbal fluency test was used to assess phonological lexicon access, which also measures executive functioning. Verbal memory, both short‐ and long‐term, was evaluated using the Babcock Memory Test. Executive functions were assessed with the Frontal Assessment Battery (FAB). The details on the demographic and neuropsychological characteristics of the two study groups are reported in the previously published study by our group (Bubbico et al. 2019). In this work, we present a reanalysis of the behavioral tests using a strategy already established by previous groups (Edmonds et al. 2015; Edmonds et al. 2016; Mapstone et al. 2014). Specifically, raw scores on each of the neuropsychological measures were first transformed into age/educational level‐corrected *z*‐scores based on the means and standard deviations of the normal reference group specific to each neuropsychological measure. Thus, one‐sample *t*‐tests against zero (i.e., T2 = T1) were used to assess the presence of a significant positive or negative delta.

### 2.3 MRI Data Collection

Participants underwent an MRI protocol before (T1) and after (T2) the FLL intervention. MR images were acquired using a Philips Achieva 3 Tesla scanner (Philips Medical Systems, Best, Netherlands). Structural MR images, obtained using a 3D fast field echo T_1_‐weighted sequence (dimensions: 170 × 240 × 240 mm^3^, slice thickness = 1 mm, in‐plane voxel size = 1 × 1 mm), were used in rs‐fMRI preprocessing to derive the grey/white/CSF masks used in the confound removal process. For each session, two runs of BOLD rs‐fMRI data were acquired with eyes closed using a gradient‐echo T_2_^*^‐weighted echo‐planar (EPI) sequence (FLL group: 3 runs; dimensions: 80 × 80 × 35 × 180; voxel size: 3 × 3 × 3 mm^3^; FOV 240 mm; TR = 1.8 s, Sham group: dimensions: 64 × 64 × 30 × 145; voxel size: 4 × 4 × 4 mm^3^; FOV = 256 mm; TR = 1.67 s). The participants were awake and responsive during the MRI session, as periodically verified by asking them to make a small movement with their right foot in the time intervals between different acquisition runs.

### 2.4 Rs‐fMRI Data Analysis

The data analysis was carried out using the Conn toolbox, version 22 (https://web.conn‐toolbox.org), in a standard pipeline employing default options (Whitfield‐Gabrieli and Nieto‐Castanon 2012). Functional images were realigned using SPM's realign and unwarp procedure, with scans aligned to a reference image through least squares and a rigid body transformation. Temporal misalignment between different slices of the functional data, introduced by the sequential nature of the fMRI acquisition protocol, was corrected using the SPM12 slice‐timing correction (STC) procedure (Henson et al. 1999). Outliers were identified using the ART tool based on the framewise displacement or BOLD signal fluctuations. Both functional and structural data were normalized to MNI space, segmented into tissue types (gray matter, white matter, CSF), and resampled to 2 mm isotropic voxels. Finally, functional data were smoothed with an 8 mm Gaussian kernel to enhance the BOLD signal‐to‐noise ratio and reduce the influence of residual variability in functional signal and gyral anatomy across subjects (Nieto‐Castanon 2020). Functional data were denoised using a standard pipeline, which included the removal of confounding variables. Specifically, white matter and CSF noise were defined from the observed BOLD signal within two anatomically defined noise regions, created by applying a one‐voxel binary erosion to the white matter and CSF posterior probability maps. Five noise components were estimated within each region, including the average BOLD signal and the first four components derived from principal component analysis (PCA). In addition, twelve noise components were defined from the estimated subject motion parameters, including three translation and three rotation parameters, along with their first‐order derivatives, to minimize motion‐related variability in the BOLD signal (Chai et al. 2016). The BOLD time series was then bandpass filtered (0.008–0.09 Hz). A component‐based noise correction (CompCor) noise components in white matter and CSF were computed by extracting the mean BOLD signal and principal components orthogonal to it, along with motion parameters and outliers, within eroded segmentation masks (Behzadi et al. 2007). Seed‐based connectivity analysis was performed by calculating the Fisher‐transformed correlation coefficients between each seed region and all other brain voxels. This analysis was performed for 26 cerebellar subregions, defined by the Conn‐integrated automated AAL‐3 (Rolls et al. 2020), including the left and right Crus I and II; the left and right Lobules III, IV/V, VI, VIIB, VIII, IX, and X in the cerebellar hemispheres; and Lobules I‐II, III, IV/V, VI, VII, VIII, IX, and X in the Vermis, as in Delli Pizzi et al. (2024).

At the voxel‐wise level, resting‐state fMRI analyses were conducted using a General Linear Model (GLM) approach. Each voxel underwent individual GLM estimation, with the first‐level connectivity measure (expressed as the difference in z‐Fisher transformed connectivity strength between post‐treatment (T2) and pre‐treatment (T1) conditions) serving as the dependent variable and the two groups (FLL and sham) as independent variables. Age and educational level were included as nuisance factors. Hypotheses at the voxel level were tested using multivariate parametric statistics with random effects across subjects and sample covariance estimation across multiple measures. Cluster‐level inferences (for contiguous voxel groups) were derived from parametric statistics using Gaussian Random Field theory (Worsley et al. 1996). Results were thresholded using a combination of a voxel‐level cluster‐forming threshold of *p* < 0.001 and a false discovery rate (FDR)‐corrected cluster‐size threshold of *p* < 0.05 (Chumbley et al. 2010). For a more comprehensive network‐level analysis, we parceled the z‐maps originating from each cerebellar seed showing FLL‐induced changes at the voxel‐wise level according to Yeo's seven networks parcellation (Yeo et al. 2011) and extracted the *z*‐scores from each entire network. Between‐group comparisons (FLL vs. Control) were conducted using ANCOVA, with age and education level as covariates. Bonferroni's correction was applied to account for multiple comparisons (corrected *p* = 0.05/7 networks/2 hemispheres = 0.004).

### 2.5 Spatial Overlaps Between Rs‐fMRI and Receptor Density Maps

A correlation analysis was performed to investigate the spatial overlap between the significant changes induced by FLL on the rs‐fMRI maps (derived from the seed‐based analysis described above) and PET‐based maps available at https://github.com/netneurolab/hansen_receptors (Hansen et al. 2022).

Specifically, distinct neuroreceptor systems were examined using various radiotracers: [^11^C]Flumazenil, a GABA tracer, acquired from 16 subjects (mean age 26.6 ± 8) (Nørgaard et al. 2021); [^11^C]OMAR, which targets the CB_1_ cannabinoid receptor, from 77 subjects (mean age 30.0 ± 8.9) (Normandin et al. 2015); and [^11^C]ABP688, which binds to the mGluR_5_ receptor. This tracer was analyzed, and their measurement were averaged across multiple cohorts, including 22 subjects (mean age 67.9 ± 9.6) (Rosa‐Neto and Kobayashi, as reported in Hansen et al. 2022), 28 subjects (mean age 33.1 ± 11.2) (DuBois et al. 2016), and 73 subjects (mean age 19.9 ± 3.04) (Smart et al. 2019). *Z*‐scores, reflecting the strength of rs‐FC, were extracted for each region of the Schaefer Atlas (100 components, 7 networks). To address the issue related to the spatial autocorrelation in brain maps, the BrainSMASH technique (Burt et al. 2020), available in the neuromaps package (https://github.com/netneurolab/neuromaps), was employed. This approach generates a set of 1000 null receptor maps that preserve the spatial autocorrelation of the original data, maintaining the same mean and variance. Each correlation was recalculated using these null maps to create a null distribution, from which a *p* value accounting for spatial autocorrelation was derived. Moreover, we further applied a Bonferroni's correction to BrainSMASH outputs tested in the left hemisphere, where connectivity changes induced by FLL occurred (corrected *p* = 0.05/4 receptor PET maps = 0.0125).

## 3 Results

### 3.1 Cognitive Assessment Before and After Treatment

The acquisition of a second language significantly influenced general cognitive functions (Table 1), as assessed by the MMSE (*p* < 0.001), as well as delayed verbal memory, assessed by the Babcock Story Recall Test (*p* = 0.009). No other cognitive domain showed a significant pre/posttreatment effect.

**TABLE 1:** Demographic and neuropsychological features of the study cohorts.

Test	Mean	SD	t13	*p* value
MMSE	1.715	1.15993	5.532	***p* < 0.001**
TMT A	−0.34	0.76856	−1.655	0.122
TMT B	−0.3007	1.57947	−0.712	0.489
TMT B‐A	−0.7707	4.23363	−0.681	0.508
IR BAB	0.0886	0.37986	0.872	0.399
DR BAB	0.0664	0.08149	3.05	**0.009**
FAS	0.0129	0.05355	0.898	0.385
FAB	−0.17	0.38262	−1.662	0.12

*Note*: Significant results are reported in bold. The positive z‐values are attributable to a greater effect at T2 compared to T1, indicating an improvement in performance for both the MMSE and Babcock tests. In fact, the higher the scores, the better the performance.

Abbreviations: BAB‐DR: Babcock Test—Delayed Recall, FAB: Frontal Assessment Battery; FAS: Verbal Fluency Test; IR: Immediate Recall,

MMSE: Mini‐Mental State Examination; TMT: Trail Making Test.

### 3.2 Voxel‐Wise Rs‐FC Changes Within the Cerebellar‐Neocortical Circuit

**FIGURE 1:**
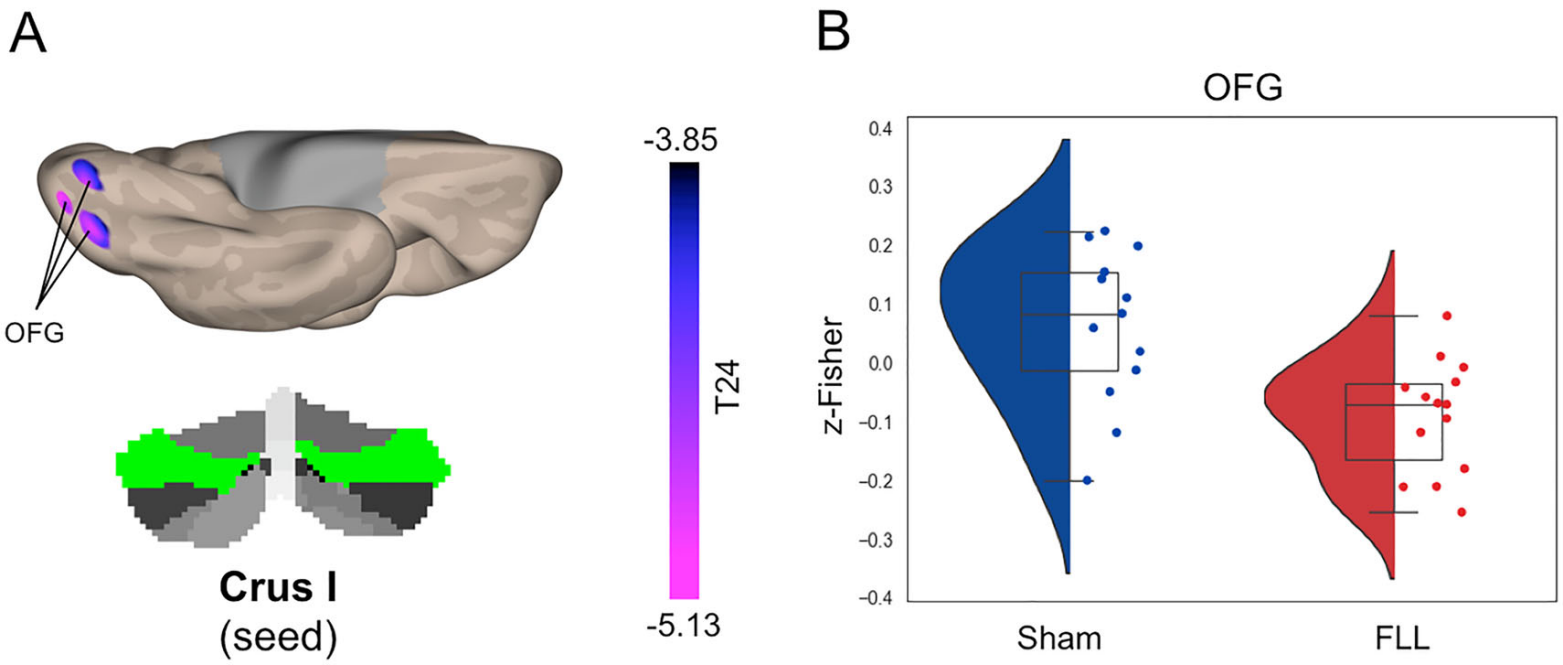
**FLL‐induced changes in Crus I functional Connectivity**. Panel A shows the surface unthresholded cortical map illustrating the topographic distribution of FLL‐induced changes in functional connectivity on the medial surface of the left hemisphere. Violet‐blue clusters indicate decreased functional connectivity. The seed location for the seed‐to‐voxel functional connectivity analysis, highlighted in green, is shown at the bottom. Panel B reports the violin plots showing the group distribution (red: FLL; blue: Sham) of functional connectivity within the significant cluster. The box shows the quartiles of the dataset while the whiskers indicate the rest of the distribution, represented as multiple (by default, as in this case: 1.5) of the “inter‐quartile range” (IQR), which is the range between the lower and upper quartile covered by the inner box. The IQRs function evaluates observations outside this range as potential “outliers”, which are then displayed outside the whiskers.

**FIGURE 2:**
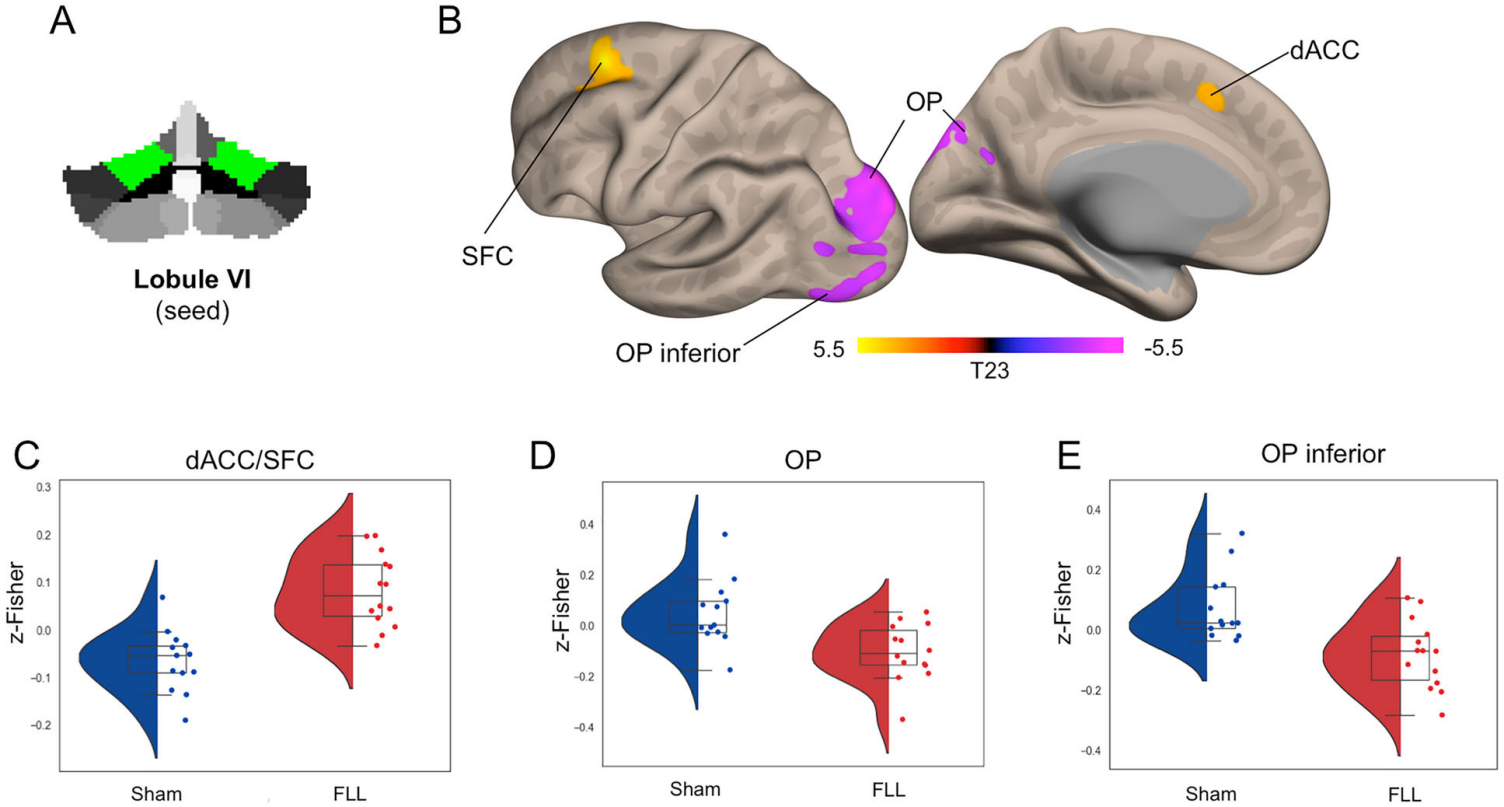
**FLL‐induced changes in Lobule VI functional connectivity**. Panel A shows the seed location for the seed‐to‐voxel functional connectivity analysis. Panel B shows the surface unthresholded cortical maps illustrating the topographic distribution of FLL‐induced changes in functional connectivity on the lateral and medial surfaces of the left hemisphere. Yellow‐red and violet‐blue clusters indicate increased or decreased functional connectivity, respectively. Panels C–E report the violin plots showing the group distribution (red: FLL; blue: Sham) of functional connectivity within the significant clusters.

**FIGURE 3:**
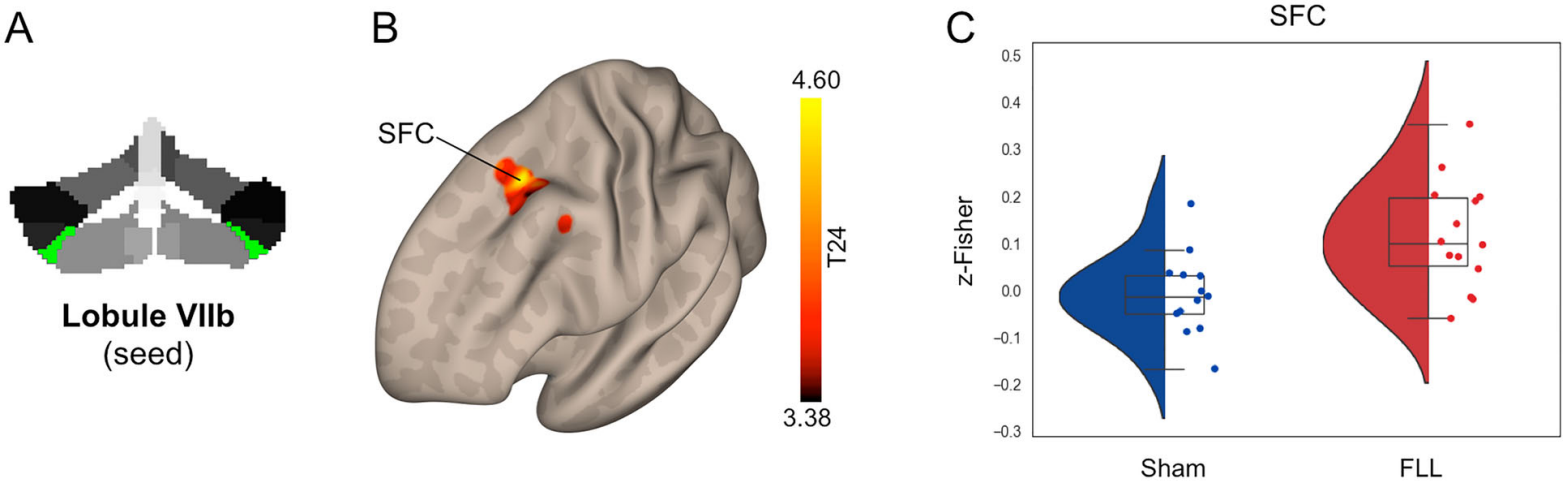
**FLL‐induced changes in Lobule VIIb functional connectivity**. Panel A shows the seed location for the seed‐to‐voxel functional connectivity analysis. Panel B shows the surface unthresholded cortical maps illustrating the topographic distribution of FLL‐induced changes in functional connectivity on the lateral surface of the left hemisphere (frontal view). Yellow‐red clusters indicate increased functional connectivity. Panel C reports the violin plots showing the group distribution (red: FLL; blue: Sham) of functional connectivity within the significant cluster.

**FIGURE 4:**
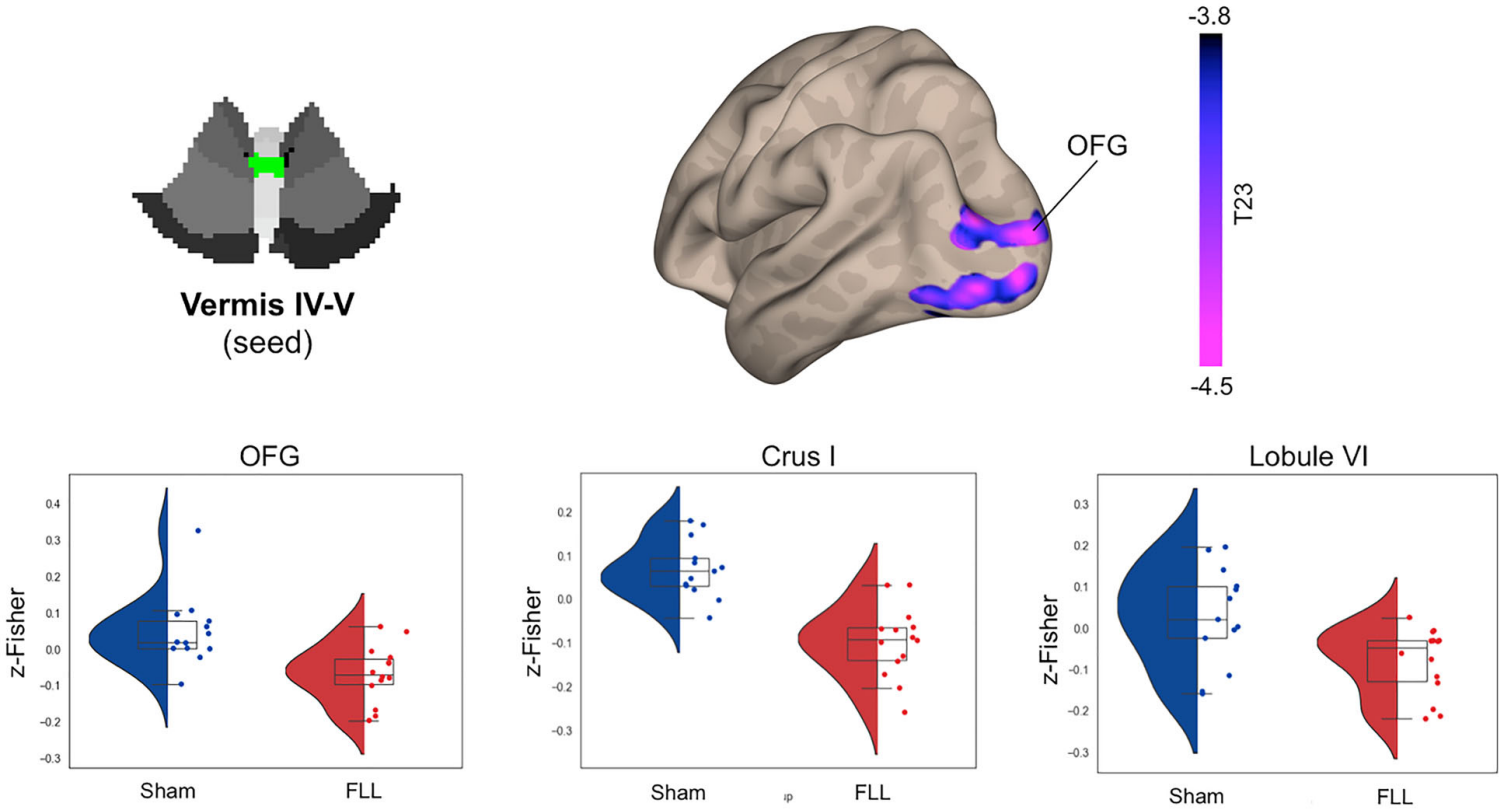
**FLL‐induced changes in Vermis IV‐V functional connectivity**. Panel A shows the seed location for the seed‐to‐voxel functional connectivity analysis. Panel B shows the surface unthresholded cortical maps illustrating the topographic distribution of FLL‐induced changes in functional connectivity on the lateral surface of the left hemisphere (posterior view). Violet‐blue clusters indicate decreased functional connectivity. Panel C reports the violin plots showing the group distribution (red: FLL; blue: Sham) of functional connectivity within the significant clusters.

Whole‐brain voxel‐wise analysis revealed that the FLL group, compared to the sham group, showed selective changes in the cortical functional connectivity of four cerebellar subregions: Crus I, Lobule VI, Lobule VIIb, and Vermis IV/V (Table 2). Specifically, the FLL group exhibited decreased connectivity between Crus I and the occipital fusiform gyrus (OFG; Figure 1). The FLL group showed increased connectivity between Lobule VI/Lobule VIIb and the superior frontal cortex (SFC) (Figures 2 and 3). Lobule VII showed increased connectivity with the dorsal anterior cingulate cortex (dACC) and decreased connectivity with the occipital pole (OP) (Figure 3). Finally, the FLL group displayed decreased connectivity between Vermis IV/V and the OP, as well as with Crus I and Lobule VI (Figure 4). No significant relationship was found between pre/post changes in the resting‐state fMRI data and neuropsychological outcomes.

**TABLE 2:** Significant clusters obtained from seed‐based analyses and their characteristics/location.

Seed	Cluster	MNI coordinates			Size	Size FWE	Size FDR	Size *p*‐uncorr	Peak FWE	Peak p‐uncorr	CI (inferior/superior)	Effect size (Eta‐squared)
		*X*	*Y*	*Z*								
Crus I	L‐OFG	−26	−84	−18	161	0.04017	0.04922	0.000019	0.639995	0.000019	HC: −0.0172/0.1416 FLL: −0.1465/−0.0363	0.539 (large)
Lobule VI	L‐OP	−12	−92	30	608	0.00001	0.00002	0.000001	0.116418	0.000001	HC: −0.0310/0.1271 FLL: −0.1710/−0.0407	0.620 (large)
	SFC/ dACC	−22	26	52	528	0.00004	0.00003	0.000001	0.071692	0.000001	HC: −0.1059/−0.0265 FLL: 0.0370/0.1249	0.642 (large)
	L‐iOP	−50	−84	−8	189	0.02415	0.01293	0.000072	0.931483	0.000072	HC: 0.0062/0.1417 FLL: −0.1475/−0.0155	0.561 (large)
Lobule VIIb	SFC	−22	10	52	183	0.02384	0.04050	0.000022	0.678775	0.000022	HC: −0.0622/0.0435 FLL: 0.0490/0.1828	0.565 (large)
Vermis IV‐V	L‐OP	−24	−94	16	567	0.00002	0.00002	0.000039	0.817113	0.000039	HC: −0.0125/0.1073 FLL: −0.1141/−0.0238	0.638 (large)
	Crus I	28	−70	−28	298	0.00215	0.00140	0.000001	0.092577	0.000001	HC: 0.0280/0.1079 FLL: −0.1459/−0.0522	0.684 (large)
	Lobule VI	−34	−54	−26	161	0.04451	0.01963	0.000007	0.363348	0.000007	HC: −0.0456/0.0986 FLL: −0.1289/−0.0346	0.615 (large)

Abbreviations: CI: Confidence Interval; dACC: dorsal Anterior Cingulate Cortex; iOP: Occipital Pole, inferior; L: Left; OFG: Occipital Fusiform Gyrus; OP: Occipital Pole: OP; SFC: Superior Frontal Gyrus.

### 3.3 Network‐Level Rs‐FC Changes Within the Cerebellar‐Neocortical Circuit

At the network level, FLL‐related changes involved cortical areas related to higher‐order networks, such as the Salience/Ventral Attention Network (SVAN), as well as primary sensory regions like the visual cortex. Specifically, the FLL group, compared to the sham group, showed a selective decrease in the functional connectivity of Crus I (Table S1), Lobule VI (Table S2), and Vermis IV‐V (Table S3) with the visual network. Moreover, the FLL group exhibited decreased connectivity of Lobule VIIb (Table S4) with the SVAN and increased connectivity with the FPCN, although the latter did not survive correction for multiple comparisons.

### 3.4 Spatial Overlap Between the Rs‐fMRI and Receptor Expression Maps

**FIGURE 5:**
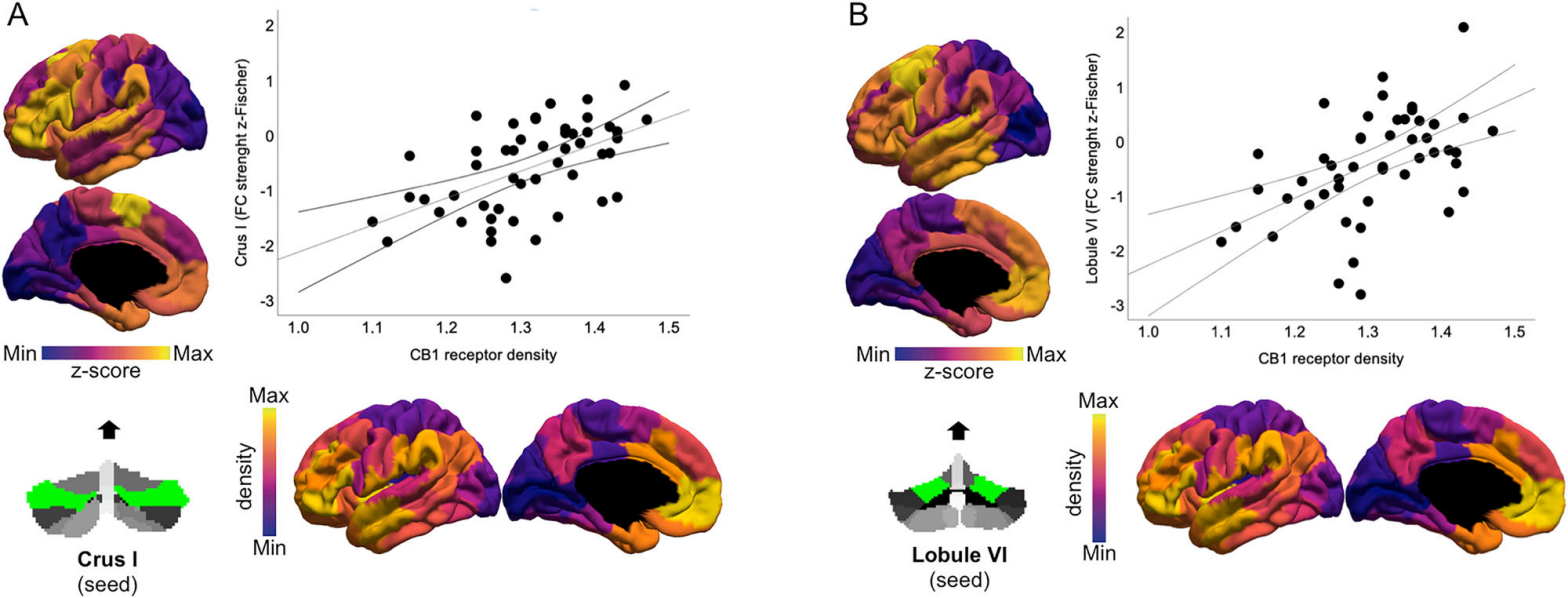
**Relationships between the connectivity modulations and receptor density**. Scatterplots of the spatial relationship between the strength of Crus I (Panel A) and Lobule VI (Panel B) functional connectivity (calculated from the comparison of T2‐T1 maps between FLL and placebo groups, with the *y*‐axis in both panels) and the expression of CB_1_ receptors (*x* axis in both panels), across the 100 Schaefer parcellation.

By evaluating the spatial correlation between rs‐FC metrics and receptor expression data from atlases, we identified a significant topographical correspondence between the rs‐FC modification of Crus I (Figure 5A; *R* = 0.534, uncorrected *p* < 0.001, SA‐corrected *p* = 0.002) and lobule VI (Figure 5B; *R* = 0.525, uncorrected *p* < 0.001, SA‐corrected *p* < 0.001) with the density of the CB_1_ receptor. Furthermore, with a trend toward significance, the distribution of mGluR_5_ receptors overlaps with the rs‐FC modification of Crus I (*R* = 0.775, uncorrected *p* = 0.002, SA‐corrected *p* = 0.02) and Vermis IV‐V (*R* = 0.420, uncorrected *p* = 0.003, SA‐corrected *p* = 0.051). Lastly, the rs‐FC modification of the Vermis IV‐V overlaps, with a trend toward significance, with the distribution of the GABA_a_ receptor (*R* = −0.322, uncorrected *p* = 0.027, SA‐corrected *p* = 0.037). No further significant overlaps were found between rs‐FC changes and the expression of other brain receptors tested (Table S5).

## 4 Discussion

The present study conducted a reanalysis of a previously published database to provide a comprehensive assessment of the modulation of cerebellar‐cortical functional connectivity induced by FLL, focusing on the contributions of distinct cerebellar regions. FLL altered the connectivity of the Crus I, Lobule VI, Lobule VIIb, and Vermis IV‐V. The changes in rs‐FC of Crus I and Lobule VI encompassed associative cortical regions rich in CB_1_ receptors. On the other hand, with a trend toward significance, the rs‐FC changes in Lobule VI and Vermis IV‐V overlapped with the topography of mGluR5 receptors, whereas the changes in Vermis IV‐V corresponded to the distribution of GABAa receptors.

In line with our previous study (Bubbico et al. 2019), the reanalysis of the neuropsychological battery revealed that the FLL had a behavioral effect, generally incrementing the scores at the MMSE and specifically improving delayed recall, as assessed by the Babcock Test—a verbal memory measure in which participants listen to a brief story and are asked to recall it both immediately and after a delay.

At the neural level, a common characteristic of the effect of FLL was the reduction of the connectivity of the Crus I, Lobule VI, and Vermis IV‐V with the visual cortex/network. On the other hand, we also observed a significant increase in connectivity between Lobule VI and VIIb and the prefrontal cortex. The neural changes observed in Crus I, Lobule VI, and Vermis IV‐V offer valuable insights into the adaptive mechanisms of the aging brain. The reduction in connectivity with the visual cortex, alongside the increase in connectivity with the prefrontal cortex, aligns with the posterior‐to‐anterior shift in aging (PASA) model (Davis et al. 2008; Dennis et al. 2008; Grady et al. 1994) and subsequent evidence (Hoffman and Morcom 2018). The PASA model suggests that, during aging, the brain compensates for physiological decline by reducing activity in posterior brain regions (such as the visual cortex) and increasing activity in anterior brain regions (such as the prefrontal cortex) (Davis et al. 2008; Dennis et al. 2008; Grady et al. 1994; Delli Pizzi et al. 2020). Specifically, in our study, the reduction in connectivity with the visual cortex in Crus I, Lobule VI, and Vermis IV‐V supports the idea that older adults rely less on sensory input for motor and cognitive processes, likely shifting the functional load to more central cortical regions (Davis et al. 2008; Dennis et al. 2008; Grady et al. 1994). In contrast, the increase in connectivity between Lobule VI, VIIb, and the area of the prefrontal cortex associated with executive control, like the SFC and the dACC, aligns with the existence of a general pattern of reciprocal connections between the cerebellar and the prefrontal cortex (Habas et al. 2021, 2009). This process may reflect a compensatory mechanism aimed at maintaining executive control and cognitive flexibility while other brain areas undergo functional decline.

We observed a spatial correlation between the rs‐FC changes in Crus I/Lobule VI and the density of CB_1_ receptors. Among the components of the endocannabinoid system, the CB_1_ receptor is widely expressed in the brain, particularly in cognitive hubs such as the hippocampus, the neocortex, and the cerebellum (Carey et al. 2011; Katona and Freund 2012). CB_1_ receptors mediate both short‐term (Kano et al. 2009) and sustained effects, such as long‐term depression of inhibition through persistent CB_1_ activation, which suppresses GABA release (Chevaleyre and Castillo 2003; Chevaleyre et al. 2007). Interestingly, aging significantly affects the endocannabinoid system, particularly CB_1_ receptor availability and coupling efficiency. Animal studies report region‐specific reductions in endocannabinoid levels and CB_1_ receptor expression during aging, particularly in the basal ganglia and cerebellum (Berrendero et al. 1998). Indeed, the activation of CB_1_ receptors is critical for protecting against age‐related cognitive decline. Animal studies revealed that the deletion of the CB_1_ receptor gene accelerates hippocampal degeneration and impairs learning and memory abilities (Bilkei‐Gorzo et al. 2005). The activation of the endocannabinoid system also inhibits reactive oxygen species production in neuronal cells (Paloczi et al. 2018), and oxidative stress increases CB_1_ receptor expression and endocannabinoid biosynthesis (Ambrozewicz et al. 2018; Matthews et al. 2016). Restoring CB_1_ signaling has been shown to reverse age‐related cognitive decline in older mice (Bilkei‐Gorzo et al. 2017; Murphy and Le Foll 2020). Thus, our results suggest a potential mechanistic process associated with CB_1_ through which FLL modifies the rs‐FC between Lobule VI and prefrontal regions. Moreover, the trend toward a significant overlap between mGluR5 receptors and the rs‐FC modification of Crus I and Lobule VI aligns with the idea that the mGluR5 receptor plays a key role in brain aging by modulating different cell types in the central nervous system (Carvalho et al. 2019), and that mGluR5 and CB_1_ receptors act in concert to activate neuroprotective cell signaling pathways and promote neuronal survival (Batista et al. 2016; Carvalho et al. 2017).

In addition, we observed a trend toward a significant spatial overlap between the expression of GABAa receptors and the reduced connectivity of the Vermis IV‐V with the visual cortex, a region with high GABAa content. GABAergic inhibition is critical for sensory processing, attention, memory, and learning. Our results fit with multiple lines of evidence supporting the role of GABAa receptors in regulating learning and memory. Deletion of extrasynaptic GABAa receptors impairs these processes (Engin et al. 2020). The number of postsynaptic GABA_a_ receptors directly impacts synaptic plasticity, with even modest reductions (5%–35%) causing significant behavioral changes (Luscher et al. 2011; Shen et al. 2010). Age‐related alterations in GABA_a_ receptor subunits, affecting channel kinetics and ligand binding, are linked to cognitive decline (Rissman and Mobley 2011). Previous studies suggest that hippocampal GABAergic loss during aging may be compensated by upregulating GABA_a_ receptors, increasing their sensitivity to GABA (Ruano et al. 2000; Vela et al. 2003). A reduction in GABAa receptors has also been observed in the brains of individuals with Alzheimer's disease (Bernareggi et al. 2007). Clinical trials show that drugs targeting specific GABA_a_ receptor subtypes improve mnemonic functions (Lewis et al. 2008). Finally, selective GABA_a_ receptor agonists like muscimol protect against amyloid β‐induced neurotoxicity in rodent neurons (Nava‐Mesa et al. 2014).

The present study has some limitations. First, FLL had a significant effect on the MMSE, which is a rudimentary scale for studying global cognitive decline. However, we did not observe a significant effect of FLL on several cognitive domains, except for the long‐term verbal memory assessed through the Babcock Story Recall Test. Therefore, the present results should be replicated with a more intense/prolonged intervention, which is expected to produce more robust effects on cognitive performance. The limited range of cognitive assessment might also cause the lack of brain‐behavioral correlations. A related issue pertains to the lack of a correlation between the imaging and behavioral results. We cannot say if the lack of a significant correlation is due to the low sample size, limiting statistical power, or the limited impact of FLL on behavioral measures. Future studies with larger samples and additional cognitive training groups could clarify this point. Regarding the issue of the limited sample size, we believe that the results of this exploratory study, while not allowing for definitive conclusions, nonetheless represent an important preliminary step toward understanding the effects of FLL on brain connectivity. Third, the use of an existing database limited our ability to preregister hypotheses and statistical analyses. Nevertheless, this analysis was motivated by recent advancements in cerebellar parcellation (Rolls et al. 2020) and receptor/transporter mapping (Hansen et al. 2022) that were unpredictable at the time of the study design. Fourth, we would like to highlight that higher education levels could have a moderating effect on both behavioral and imaging measures. In fact, a higher level of education could influence the speed of FLL and baseline cognitive performance, thereby contributing to shaping the effect of language learning on brain connectivity. These moderating effects should be considered in future studies, as they could help better clarify the role of individual variables in the language learning process and their impact on brain functions. Finally, from an imaging point of view, alignment with individual anatomy remains a challenge, and age differences in receptor maps could introduce bias. In that regard, a potential limitation of our study is the difference in age range between the PET receptor maps and the participants in our study. Specifically, the age range for CB1 receptors (30.0 ± 8.9 years) and GABAa receptors (26.6 ± 8 years) does not align with the age range of our study groups, which could introduce bias. While age differences may be less relevant for mGluR5, where specific receptor binding patterns are studied across different age groups and receptor systems—given that age‐related effects on receptor density may be less pronounced—it is still important to consider age‐related changes in the number of receptors in the neocortex, as these could influence the spatial relationships between receptors and brain circuits (Lee and Kim 2022).

In conclusion, our study indicates that FLL modulates the connectivity of specific cerebellar subregions that affect sensory and associative cortical networks. This modulation primarily aligns with the distribution of CB_1_ receptors, offering new insights into the brain mechanisms that support cognitive reserve in aging. Further investigation will be required to refine the mechanistic interpretations and verify the direct causal evidence linking these receptors to the observed changes in connectivity.

## Author Contributions

**Giovanna Bubbico**: conceptualization, funding acquisition, project administration, data curation, writing–original draft, investigation, methodology. **Federica Tomaiuolo**: formal analysis, methodology, investigation, writing–review and editing. **Carlo Sestieri**: conceptualization, investigation, writing – original draft. **Golnoush Akhlaghipour**: writing–review and editing. **Alberto Granzotto**: writing–review and editing. **Antonio Ferretti**: writing–review and editing, resources. **Mauro Gianni Perrucci**: writing–review and editing. **Stefano L. Sensi**: writing–review and editing, supervision, resources. **Stefano Delli Pizzi**: conceptualization, investigation, writing–original draft, methodology, formal analysis, supervision.

## Ethics Statement

The study received approval from the Research and Ethics Committee (Ethical approval number: Prot. Nr. 835, 24‐04‐2015), and all participants provided written informed consent. All procedures adhered to the ethical principles outlined in the Declaration of Helsinki. The authors confirm that the reproduced material has been used with the appropriate permissions and acknowledgments in accordance with copyright regulations.

## Conflicts of Interest

The authors declare no conflicts of interest.

### Peer Review

The peer review history for this article is available at https://publons.com/publon/10.1002/brb3.70565.

## Supporting information



Supporting Information

## Data Availability

The data that support the findings of this study are available upon reasonable request from the corresponding author.
