# Supplementary material for: Learning a Foreign Language in Older Adults Shapes the Functional Connectivity of Distinct Cerebellar Sub‐Regions With Cortical Areas Rich in CB1 Receptor Expression
Source: Brain Behav. 2025 May 26;15(5):e70565. doi: 10.1002/brb3.70565 (PMC12105657; doi:10.1002/brb3.70565)
Supplement: Supplementary file 1 — Supporting Information [file BRB3-15-e70565-s001.docx]

**Supplementary Table 1.** Network-level analysis of the Crus I functional connectivity changes with the neocortex.

| Yeo Network | HC | | FLL | | ANCOVA | |
| --- | --- | --- | --- | --- | --- | --- |
|  | Mean | SD | Mean | SD | F | p |
| L-FPCN | 0.013 | 0.049 | 0.014 | 0.066 | 0.004 | 0.952 |
| L-DMN | 0.027 | 0.040 | -0.002 | 0.039 | 3.436 | 0.076 |
| L-DAN | 0.018 | 0.041 | -0.021 | 0.060 | 3.667 | 0.067 |
| L-Limbic | -0.005 | 0.053 | 0.015 | 0.067 | 0.724 | 0.403 |
| L-SVAN | -0.010 | 0.060 | 0.012 | 0.061 | 0.877 | 0.358 |
| L-SMN | -0.018 | 0.054 | -0.022 | 0.048 | 0.051 | 0.823 |
| L-VIS | 0.017 | 0.052 | -0.066 | 0.057 | 15.564 | **0.001** |
| R-FPCN | -0.006 | 0.041 | -0.021 | 0.055 | 0.671 | 0.420 |
| R-DMN | 0.013 | 0.063 | -0.021 | 0.049 | 2.558 | 0.122 |
| R-DAN | -0.006 | 0.041 | -0.020 | 0.055 | 0.555 | 0.463 |
| R-Limbic | 0.002 | 0.083 | -0.015 | 0.050 | 0.412 | 0.527 |
| R-SVAN | -0.035 | 0.062 | -0.010 | 0.070 | 0.959 | 0.337 |
| R-SMN | -0.023 | 0.041 | -0.013 | 0.070 | 0.197 | 0.661 |
| R-VIS | 0.005 | 0.066 | -0.033 | 0.083 | 1.719 | 0.202 |

*Abbreviations:* DAN=Dorsal Attention Network; DMN=Default Mode Network; FPCN=Fronto-Parietal Control Network; SVAN=Salience/Ventral Attention Network; SMN=SomatoMotor Network; VIS=Visual Network.

**Supplementary Table 2.** Network-level analysis of the Lobule VI functional connectivity changes with the neocortex.

| Yeo Network | HC | | FLL | | ANCOVA | |
| --- | --- | --- | --- | --- | --- | --- |
|  | Mean | SD | Mean | SD | F | p |
| L-FPCN | -0.013 | 0.052 | 0.001 | 0.068 | 0.253 | 0.620 |
| L-DMN | -0.020 | 0.031 | -0.005 | 0.049 | 1.641 | 0.213 |
| L-DAN | 0.007 | 0.049 | -0.027 | 0.076 | 3.243 | 0.085 |
| L-Limbic | -0.006 | 0.056 | 0.036 | 0.063 | 2.023 | 0.168 |
| L-SVAN | 0.000 | 0.059 | 0.019 | 0.071 | 0.000 | 0.996 |
| L-SMN | -0.008 | 0.050 | -0.005 | 0.071 | 0.300 | 0.589 |
| L-VIS | 0.024 | 0.050 | -0.075 | 0.093 | 14.776 | **0.001** |
| R-FPCN | -0.009 | 0.048 | -0.028 | 0.060 | 0.030 | 0.865 |
| R-DMN | -0.012 | 0.051 | -0.022 | 0.081 | 0.290 | 0.595 |
| R-DAN | -0.007 | 0.046 | -0.028 | 0.056 | 0.006 | 0.938 |
| R-Limbic | 0.001 | 0.063 | -0.004 | 0.048 | 0.036 | 0.852 |
| R-SVAN | 0.005 | 0.055 | -0.025 | 0.060 | 1.468 | 0.238 |
| R-SMN | 0.017 | 0.063 | -0.013 | 0.101 | 1.320 | 0.262 |
| R-VIS | 0.025 | 0.061 | -0.029 | 0.096 | 4.231 | 0.051 |

*Abbreviations:* DAN=Dorsal Attention Network; DMN=Default Mode Network; FPCN=Fronto-Parietal Control Network; SVAN=Salience/Ventral Attention Network; SMN=SomatoMotor Network; VIS=Visual Network.

**Supplementary Table 3.** Network-level analysis of the Vermix IV-V functional connectivity changes with the neocortex.

| Yeo Network | HC | | FLL | | ANCOVA | |
| --- | --- | --- | --- | --- | --- | --- |
|  | Mean | SD | Mean | SD | F | p |
| L-FPCN | 0.008 | 0.065 | 0.022 | 0.060 | 0.589 | 0.451 |
| L-DMN | -0.002 | 0.047 | -0.010 | 0.064 | 0.047 | 0.831 |
| L-DAN | -0.002 | 0.035 | 0.013 | 0.055 | 0.318 | 0.578 |
| L-Limbic | 0.001 | 0.049 | 0.028 | 0.063 | 0.850 | 0.366 |
| L-SVAN | 0.005 | 0.058 | 0.034 | 0.060 | 0.940 | 0.342 |
| L-SMN | -0.028 | 0.045 | 0.023 | 0.055 | 4.440 | 0.046 |
| L-VIS | 0.020 | 0.057 | -0.035 | 0.045 | 10.164 | **0.004** |
| R-FPCN | 0.007 | 0.051 | -0.027 | 0.081 | 0.189 | 0.668 |
| R-DMN | -0.010 | 0.049 | -0.031 | 0.075 | 0.003 | 0.959 |
| R-DAN | 0.007 | 0.050 | -0.028 | 0.078 | 0.222 | 0.642 |
| R-Limbic | -0.005 | 0.046 | -0.021 | 0.056 | 0.072 | 0.791 |
| R-SVAN | 0.003 | 0.056 | -0.039 | 0.060 | 1.078 | 0.310 |
| R-SMN | -0.006 | 0.052 | -0.041 | 0.058 | 2.303 | 0.143 |
| R-VIS | 0.016 | 0.050 | -0.040 | 0.050 | 10.211 | **0.004** |

*Abbreviations:* DAN=Dorsal Attention Network; DMN=Default Mode Network; FPCN=Fronto-Parietal Control Network; SVAN=Salience/Ventral Attention Network; SMN=SomatoMotor Network; VIS=Visual Network.

**Supplementary Table 4.** Network-level analysis of the Lobule VIIb functional connectivity changes with the neocortex.

| Yeo Network | HC | | FLL | | ANCOVA | |
| --- | --- | --- | --- | --- | --- | --- |
|  | Mean | SD | Mean | SD | F | p |
| L-FPCN | 0.004 | 0.064 | 0.038 | 0.054 | 6.068 | 0.022 |
| L-DMN | -0.016 | 0.050 | -0.001 | 0.039 | 3.131 | 0.090 |
| L-DAN | -0.004 | 0.045 | -0.002 | 0.056 | 0.149 | 0.703 |
| L-Limbic | -0.014 | 0.056 | 0.024 | 0.057 | 1.379 | 0.252 |
| L-SVAN | 0.006 | 0.041 | 0.043 | 0.054 | 0.114 | 0.739 |
| L-SMN | -0.001 | 0.027 | -0.001 | 0.063 | 0.557 | 0.463 |
| L-VIS | -0.045 | 0.132 | -0.073 | 0.058 | 0.198 | 0.661 |
| R-FPCN | 0.024 | 0.058 | -0.009 | 0.051 | 0.571 | 0.457 |
| R-DMN | -0.002 | 0.046 | -0.016 | 0.044 | 0.010 | 0.920 |
| R-DAN | 0.025 | 0.056 | -0.012 | 0.050 | 1.001 | 0.327 |
| R-Limbic | -0.011 | 0.047 | -0.018 | 0.051 | 0.684 | 0.417 |
| R-SVAN | 0.018 | 0.064 | -0.024 | 0.051 | 13.583 | **0.001** |
| R-SMN | 0.020 | 0.047 | -0.032 | 0.065 | 7.658 | 0.011 |
| R-VIS | -0.013 | 0.068 | -0.057 | 0.059 | 1.604 | 0.218 |

*Abbreviations:* DAN=Dorsal Attention Network; DMN=Default Mode Network; FPCN=Fronto-Parietal Control Network; SVAN=Salience/Ventral Attention Network; SMN=SomatoMotor Network; VIS=Visual Network.

**Supplementary Table 5.** BrainSMASH outputs tested in the left hemisphere, where connectivity changes induced by FLL occur.

| Receptors | Crus I | | Lobule VI | | Lobule VIIb | | Vermis IV-V | |
| --- | --- | --- | --- | --- | --- | --- | --- | --- |
|  | Spatially  naive  p-value | SA corrected  p-value | Spatially  naive  p-value | SA corrected  p-value | Spatially  naive  p-value | SA corrected  p-value | Spatially  naive  p-value | SA corrected  p-value |
| CB1 | **p<0.001** | **0.002** | **p<0.001** | **p<0.001** | 0.142 | 0.308 | 0.015 | 0.128 |
| GABAa | 0.199 | 0.234 | 0.436 | 0.461 | 0.251 | 0.305 | 0.027 | 0.037 |
| mGluR5 | 0.002 | 0.02 | 0.003 | 0.051 | 0.385 | 0.577 | 0.028 | 0.213 |
| NMDA | 0.898 | 0.898 | 0.722 | 0.783 | 0.991 | 0.995 | 0.83 | 0.887 |
